# Association of infant and child health characteristics with the hazard of any medical condition or disability in Australian children

**DOI:** 10.1186/s13690-022-00913-3

**Published:** 2022-06-23

**Authors:** Kabir Ahmad, Syed Afroz Keramat, Nusrat Jahan Sathi, Enamul Kabir, Rasheda Khanam

**Affiliations:** 1 School of Business, Faculty of Business, Education, Law and Arts, University of Southern Queensland, Toowoomba, Australia; 2 Present Address: School of Business, Faculty of Business, Education, Law and Arts, and, Centre for Health Research, University of Southern Queensland, Toowoomba, Australia; 3 Economics Discipline, Social Science School, Khulna University, Khulna, 9208 Bangladesh; 4 Statistics Discipline, Khulna University, Khulna, 9208 Bangladesh; 5 School of Sciences, Faculty of Health, Engineering and Sciences, University of Southern Queensland, Toowoomba, Australia

**Keywords:** Medical condition, Disability, Hazard rate, Birthweight, Obesity, Intensive care unit

## Abstract

**Background:**

The incidence of any medical condition (e.g., sight, hearing, and speech problems, blackouts, chronic pain etc.) or disability (e.g., limited use of arms or fingers, legs, and feet, or other physical long-term health condition limiting everyday activities etc.) have been increasing among Australian children in recent decades.

**Objectives:**

This study assessed whether infant or child health characteristics might be predictors of subsequent medical conditions or disabilities in children in the first 15 years of life.

**Methods:**

Using time to event data of 5107 children, obtained from the Birth cohort of the Longitudinal Study of Australian Children, the study estimated the incidence of any medical condition or disability using the survival analysis technique. This study followed up the children from birth to 14 or 15 years of age (2004–2018) and assessed the association of infant and child health characteristics (birthweight, gestational age, use of intensive care unit or ventilator during their neonatal age and obesity) with hazard of any medical condition or disability using the random effect parametric survival regression model. The infant characteristics were measured in the Wave 1 while the children were aged 0/1 year and obesity characteristics were measured longitudinally over all the waves up to 14/15 years of age.

**Results:**

The hazard rate of any medical condition or disability for all participants was 26.13 per 1000 person-years among children in Australia. This hazard incidence rate was higher among low birthweight (39.07) children compared to the children of normal birthweight (24.89) children. The hazard rate also higher among obese (34.37) children compared to the normal weight children (24.82) and among those who had received after-birth ventilation or intensive care unit emergency services (36.87) compared to those who have not received these services (24.20). The parametric panel regression model also suggests that children with low birthweight were 1.43 times (Hazard Ratio: 1.43, 95% Confidence Interval: 1.05–1.94) more likely to have any medical condition or disability than children with normal birthweight. The time to event analyses also revealed that being recipient of after-birth emergencies (HR: 1.47, 95% CI: 1.23–1.75), being male children (HR: 1.30, 95% CI: 1.14–1.48) or being obese (HR: 1.38, 95% CI: 1.07–1.79) significantly increased the likelihood of the incidence of a medical condition or disability among children. The regression model was adjusted for socio-demographic characteristics of children and mothers..

**Conclusions:**

The study findings suggest that infants with low birth weight, hospital emergency service use and children with obesity would benefit from additional health care monitoring to minimize the risk of any medical condition or disability.

## Background

Medical conditions or disabilities generate a disease burden for children worldwide. The term ‘medical condition or disability’ refers to any disabilities and related medical conditions of adults and children, for example, sight, hearing, and speech problems, blackouts, chronic pain, nervousness, head injuries, difficulty in breathing, learning difficulties, limited use of arms or fingers, legs, and feet, gripping problem, or other physical long-term health conditions [1]. The Global Burden of Disease study in 2004 estimated that a total of 5.1% (93 million) children aged 14 or under lived with moderate or severe disability, amongst 0.7% (13 million) survived with severe medical condition [2]. In addition, approximately 150 million children aged 18 years or under had medical condition or disability, most of whom live with the reality of exclusion in the world [3]. A similar picture has been observed in Australia. In 2018, nearly 7.7% (357,500) of children under 15 years had experienced any medical condition or disability, of which 4.5% and 1.6% had severe and moderate/mild conditions, respectively [4]. In addition, the proportion of children with disability has been increased from 6.9% (295,900) in 2012 [4].

The available literature shows that children’s health state, such as having any medical condition or disability, in childhood depends on the health characteristics of the mother during pregnancy and of the infants [5–9]. Low birth weight and shorter gestation period/preterm birth are significantly associated with increased risk of any medical condition among children [7, 8, 10, 11]. Past studies in the US and China revealed that sex, a biological factor, substantially impacted disability [7, 9]. A systematic review confirmed that the likelihood of being overweight and obese increased the risk of acquiring disabilities in children by 1.54 and 1.80 times, respectively [6]. Two earlier studies had found that perinatal factors, such as low birth weight and premature birth, were associated with a higher likelihood of disability acquisition [12, 13].

Besides the biological risk factors, there are psychological and sociodemographic risk factors which are associated with hazard of any medical condition or disability [14, 15]. For example, prevalence of chronic pain was higher among people who are not in the employment or have lower income than those who are employed or have higher income [14]. Ethnicity and cultural background are other predictors of long-term medical conditions or disabilities [14]. This study intends to assess the longitudinal developmental origins of health and disease among children and hence provide importance to investigate the associations of biological risk factors from infancy and childhood characteristics with the acquisition of long-term medical conditions or disabilities in children over time.

However, one of the main limitations of the existing literature is that most of the previous studies that have provided evidence of different biological risk factors, including birthweight and obesity, for chronic conditions or disability acquisition are conducted among adults and older people [16, 17]. Though a small number of child health focused studies are available, these are either cross-sectional or not of contemporary birth cohorts [7, 8, 10]. Further, neither of earlier studies had the scope for, nor willing to investigate both birthweight and obesity factors in a single study. It is important to consider both variables because children with normal birth weight may sometimes be obese at any time point over the period of 14/15 years. Another limitation is that there is no longitudinal time to event studies or survival analyses in the Australian setting to identify the risk factors of medical condition or disability among children.

To overcome the limitations of current literature and to investigate the developmental origins of health and disease, the present study hypothesized that infant and child health-related characteristics (birthweight, obesity over childhood, gestational age and emergency service use after birth) are associated with any medical condition or disability acquisition in Australian children. This cohort study will reveal knowledge, as an early warning for the children before they enter into their adulthood, about the risk factors of the incidence of any medical condition or disability using 15-year of follow-up data from the nationally representative Australian birth cohort.

## Methods

### Data source and sample selection

The data came from the birth cohort of the Longitudinal Study of Australian Children (LSAC), a representative household survey of Australian children that began in 2004 and biennially collects information on the health (physical and socio-emotional) and development of Australian children based on the context of the bio-ecological framework of human development [18]. For the current analysis, we used Wave 1 as the baseline (*n* = 5107) and followed the development of the children up to Wave 8 (*n* = 3127) which resulted in 51,009 person-year data for the survival analysis. As this was a birth cohort, we considered all the children of the baseline survey as being independent of any medical condition or disability. Demographic characteristics, any medical condition or disability and other health related status for children were all identified using the data dictionary of the LSAC study.

### Outcome variable

The diagnostic of having long-term medical condition or disability was acquired by the LSAC study from the caregivers of the children at the survey of each wave, conducted between 2004 and 2018. Long-term medical condition or disability includes the incidence of long-term medical conditions or disabilities that have lasted or are likely to last for six months or more and restricts physical activity or physical work, as reported by the caregivers of the study children or by the study children themselves if they are aged 14 years or over. These long-term medical conditions or disabilities include any of the following 16 conditions: sight problems, hearing problems, speech problems, blackouts, difficulty learning, limited use of arms or fingers, difficulty gripping, limited use of legs and feet, other physical condition, disfigurement or deformity, shortness of breath or breathing difficulties, chronic or recurring pain or discomfort causing restriction, nervous condition causing restriction, head injuries and long-term effects as a result of head injury, stroke or other brain damage causing restriction, other long-term conditions causing restriction, or other long-term treated conditions such as arthritis, asthma, heart disease, Alzheimer’s disease, dementia etc. Based on the knowledge of healthcare systems in Australia, this study assumes that the medical conditions or disabilities reported by the respondents were identified or treated by a General Practitioner or specialist earlier. A dichotomous variable was generated and coded with the value 1 for having any of these medical conditions or disabilities and 0 for not having any of these diseases at each wave’s survey. Afterwards, time to event data were generated from the longitudinal data of all waves using the survival function to estimate the incidence rate of any health condition or disability.

### Independent variables

Based on the existing literature, as mentioned in the background section [6–9, 11–13, 16, 17], the following variables were considered as the independent variables in this study: birthweight (low: < 2500 grams, normal: 2500 – 4000 grams, high: > 4000 grams), gestational age (early: < 37 weeks, on-time: 37–41 weeks, late: > 41 weeks), obesity status (underweight: <  = 5th percentile, normal: 6th to 84th percentile, overweight: 85th to 94th percentile, obese: >  = 95th percentile) of the children and after birth emergency service use (yes: ventilation or intensive care unit service use, no: none of these services use). The options of the responses of the selected variables were available in the LSAC data as mentioned in the brackets of the variables. Birthweight, gestational age and emergency service use were measured in the Wave 1 while the children were aged 0/1 year. Obesity status of the children were measured longitudinally over all the waves from the children’s birth to 14/15 years of age.

### Control variables

This study considered the following socio-demographic covariates as confounding variables: (i) age of the children, (ii) sex of the children (male or female), (iii) whether English is spoken at home (yes or no), (iv) whether children have both parents (both parents, single parent), (v) indigenous status (yes or no), (vi) education of mother (year 12 or less, certificate, graduate degree/diploma, postgraduate), (vii) remoteness of the family residence based on the accessibility of metropolitan and regional services (highly accessible, accessible/moderately accessible, or remote/very remote).

The health indicators of this longitudinal study of birth cohort were observed and measured bi-annually with varying age of children by 1 year. For example, at Wave 1 children were aged 0 to 1, and subsequently at Wave 2 to Wave 8, children were aged 2–3 to 14–15 years old. Hence, the measurement of survival time from the measured target diseases of this study have been affected by age due to inclusion of both younger and older child in each Waves. Hence, we controlled for age for eliminating the additional burden on survival rate due to older aged children.

### Statistical analyses

Descriptive statistics were used to summarize characteristics of children and mothers. The occurrence of any health condition or disability was estimated from 51,009 person-year survival data which was derived from 5107 children followed up until 15 years age. The panel data parametric hazard model was used to estimate the hazard ratio of developing any medical condition or disability and the accumulation of incidence rate of the hazard among the participants. The random effects panel data method of survival analysis was employed to assess the impact of both time-varying and non-time variant independent variables on the occurrence of any health condition or disability. We defined the onset of having any health condition or disability as the point of time from which a child was identified having a health condition or disability by the caregiver in the follow-up surveys; and calculated the onset of time by calculating the age of the children during the reporting time. A person was denoted as being censored if they were dropped out from any point of the follow-up survey. Panel regression hazard model assumptions were checked, and the multivariate model was fit adjusting for the following confounders: age of the child during survey of each wave, sex of the child, mother’s age at childbirth, mother’s education, remoteness of residence, language spoken at home, indigenous status of children and whether the study children have both parents at home. All the confounders were checked for multi-collinearity along with the variance inflation factor (VIF) test, and no multi-collinearity was found. A predictor was considered statistically significant if the respective p-value of a particular exposure was less than or equal to 0.05 in the multivariate regression analyses. Analyses were performed using Stata version 16 (Stata Inc.).

## Results

### Study participants

**Fig. 1:**
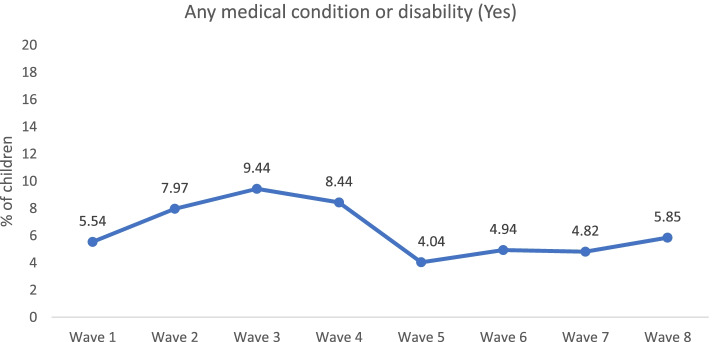
Prevalence of any medical condition or disability health hazard among Australian children across the waves, 2004–2018

Table 1 shows the participants’ baseline characteristics in Wave 1, and the representation of the baseline characteristics in the subsequent follow-ups up to Wave 8. Among the 5107 participants at baseline, 12.81% children had birthweight over 4000 grams, 5.68% of children’s weight was over 95 percentiles, 6.57% children had premature birth and 16.86% children needed ventilation/intensive care unit support after their birth. During the 15-year follow-up period, 1980 participants dropped out or were lost to follow-up. Among these dropouts, there were no significant differences in terms of characteristics of gender, birthweight, gestational age and afterbirth emergency. In Wave 8, a total of 3127 children participated; among them 51.36% were male participants, 9.35% children were overweight, 6.36% children were obese, 5.86% children had a premature birth and 16.37% children required ventilation/intensive care unit intervention after birth. The prevalence of any medical condition or disability varied longitudinally among the children, ranged in between 4.04% to 9.44% and reached to its peak during Wave 3 (9.44%) and Wave 4 (8.44%), while the children were four to seven years old (see Fig. 1).

**Table 1:** Baseline characteristics of the first wave and the subsequent follow-ups, LSAC study, 2004–2018

	Baseline	Subsequent follow-ups							Baseline characteristics of dropped-out subjects
	Wave 1, 2004 (*n* = 5107)	Wave 2, 2006 (*n* = 4606)	Wave 3, 2008 (*n* = 4386)	Wave 4, 2010 (*n* = 4242)	Wave 5, 2012 (*n* = 4085)	Wave 6, 2014 (*n* = 3764)	Wave 7, 2016 (*n* = 3381)	Wave 8, 2018 (*n* = 3127)	Dropouts/Loss to follow-up (W2-W8, *n* = 1980)
Baseline characteristics	Percent	Percent	Percent	Percent	Percent	Percent	Percent	Percent	Percent
**Sex**									
Female	48.93	49.00	48.68	48.44	48.69	48.75	48.71	48.64	49.39
Male	51.07	51.00	51.32	51.56	51.31	51.25	51.29	51.36	50.61
**Birthweight**									
Low (< 2500 grams)	5.76	5.25	5.40	5.45	5.26	5.34	5.41	5.15	6.72
Normal (2500-4000 grams)	81.44	81.44	81.3	81.19	80.95	80.9	80.83	80.84	82.37
High (>4000 grams)	12.81	13.31	13.29	13.37	13.78	13.76	13.75	14.01	10.91
**Weight percentile status**									
Underweight, < 15%	6.17	5.19	6.16	4.95	4.68	6.22	6.15	5.24	6.77
Normal weight, 15%-85%	79.05	70.67	69.97	74.3	71.06	65.75	64.21	62.49	80.51
Overweight, 85%-95%	8.44	17.85	17.01	13.79	16.03	17.48	18.07	19.06	7.07
Obese, > 95%	5.64	4.47	5.45	5.52	6.10	5.47	5.29	6.81	4.55
Missing	0.70	1.82	1.41	1.44	2.13	5.07	6.27	6.40	1.11
**Gestational age**									
Premature birth, < 37 weeks	6.57	6.39	6.44	6.35	6.28	6.28	6.07	5.86	7.70
On time birth, 37–42 weeks	88.86	89.1	89.16	89.07	89.19	89.2	89.63	89.31	88.15
Late birth, 42 + weeks	4.57	4.50	4.41	4.58	4.54	4.52	4.30	4.84	4.15
**After birth emergency**									
No emergency	83.14	83.41	83.68	83.57	83.94	83.53	83.82	83.63	82.37
Ventilation/Intensive Care Unit required	16.86	16.59	16.32	16.43	16.06	16.47	16.18	16.37	17.63
**Age of mothers at birth**									
< = 18 years	1.17	0.89	0.87	0.73	0.66	0.50	0.41	0.35	2.47
19–34 years	72.41	71.69	71.34	71.52	71.14	70.75	70.10	69.84	76.46
> = 35 years	26.41	27.42	27.79	27.75	28.2	28.75	29.49	29.80	21.06
**Have both parents**									
*Yes*	90.54	89.58	88.42	87.08	85.78	84.56	82.17	81.74	84.09
**Maternal education**									
< 12 years of education	31.72	30.37	29.72	29.64	29.14	27.82	26.74	26.12	40.60
12 years of education	25.60	25.39	25.11	24.64	24.48	24.39	23.84	23.82	28.43
Graduate/Diploma	35.59	36.72	37.59	38.12	38.59	39.73	40.85	41.20	26.71
University Masters	7.08	7.52	7.58	7.60	7.79	8.06	8.56	4.87	4.26
**Remoteness of residence**									
Highly accessible	55.46	55.63	55.29	55.15	54.64	55.03	54.67	55.19	55.89
Accessible	23.53	23.57	23.82	23.69	24.36	23.87	24.16	23.88	22.98
Moderately accessible	16.64	16.49	16.63	16.97	16.79	16.97	17.02	16.58	16.73
Remote/very remote	4.38	4.30	4.26	4.19	4.21	4.13	4.16	4.36	4.40

### Hazard rate of any medical condition or disability

The hazard rate of any medical condition or disability (at least one) for all participants was 26.13 (95% CI: 24.77–27.57) per 1000 person-years in children followed-up from age 0 to 15 years in Australia, in the period between 2004 and 2018. The hazard rate was higher for those who had low birth weight (< 2500 grams) in 2004 compared with normal birthweight children (HR: 39.07, 95% CI: 32.15–47.95; and HR: 24.89, 95% CI: 23.43–26.45 per 1000 person-years, respectively). This pattern was also observed among the underweight and obese children; among them hazard rate was around 1.5 times higher compared with normal weight children (Table 2). Table 2 also presents the incidence rate (of hazards) by gestational age and after-birth emergency service categories. Children who were born early and received after-birth ventilation or intensive care unit (ICU) services had higher hazard rate (around 1.5 times) relative to those who born on time and did not receive any emergency services, respectively.

**Table 2:** Hazard rate of any medical condition or disability in the living per 1000 person-years in children followed-up from age 0 to 15 years in Australia, according to birthweight, gestational age and obesity, 2004–2018

	**Total**	
	Hazard Rate	95% CI
**All participants**	26.13	24.77–27.57
**Birth weight**		
*Low (*< *2500 grams)*	39.07	32.15–47.49
*Normal (2500–4000 grams)*	24.89	23.43–26.45
*High (*> *4000 grams)*	28.89	25.08–33.28
**Gestational age**		
*Early (*< *37 weeks)*	35.16	29.92–44.38
*On-time (37–41 weeks)*	25.58	26.13–29.33
*Late (*> *41 weeks)*	25.24	21.49–35.65
**Obesity**		
*Underweight (*< = *5*^*th*^* Percentile)*	36.42	29.99–44.23
*Normal (6*^*th*^* to 84*^*th*^* Percentile)*	24.82	23.24–26.50
*Overweight (85*^*th*^* to 94*^*th*^* Percentile)*	26.23	22.94–29.99
*Obese (*> = *95*^*th*^* Percentile)*	34.36	28.05–42.11
**After birth emergency**		
*No*	24.20	22.78–25.71
*Yes (Ventilation/ICU)*	36.87	32.85–41.40

Any medical condition/disability includes the incidence of long-term medical condition/disability and disability limiting everyday activities; Long-term medical condition/disability includes sight problems, hearing problems, speech problems, blackouts, difficulty learning, limited use of arms or fingers, difficulty gripping, limited use of legs and feet, other physical condition, or other disfigurement which lasted for six months or more; Disability limiting every day activities includes difficulty breathing, chronic pain, nervous condition, head injury, other long-term condition, or other treated condition

**Fig. 2:**
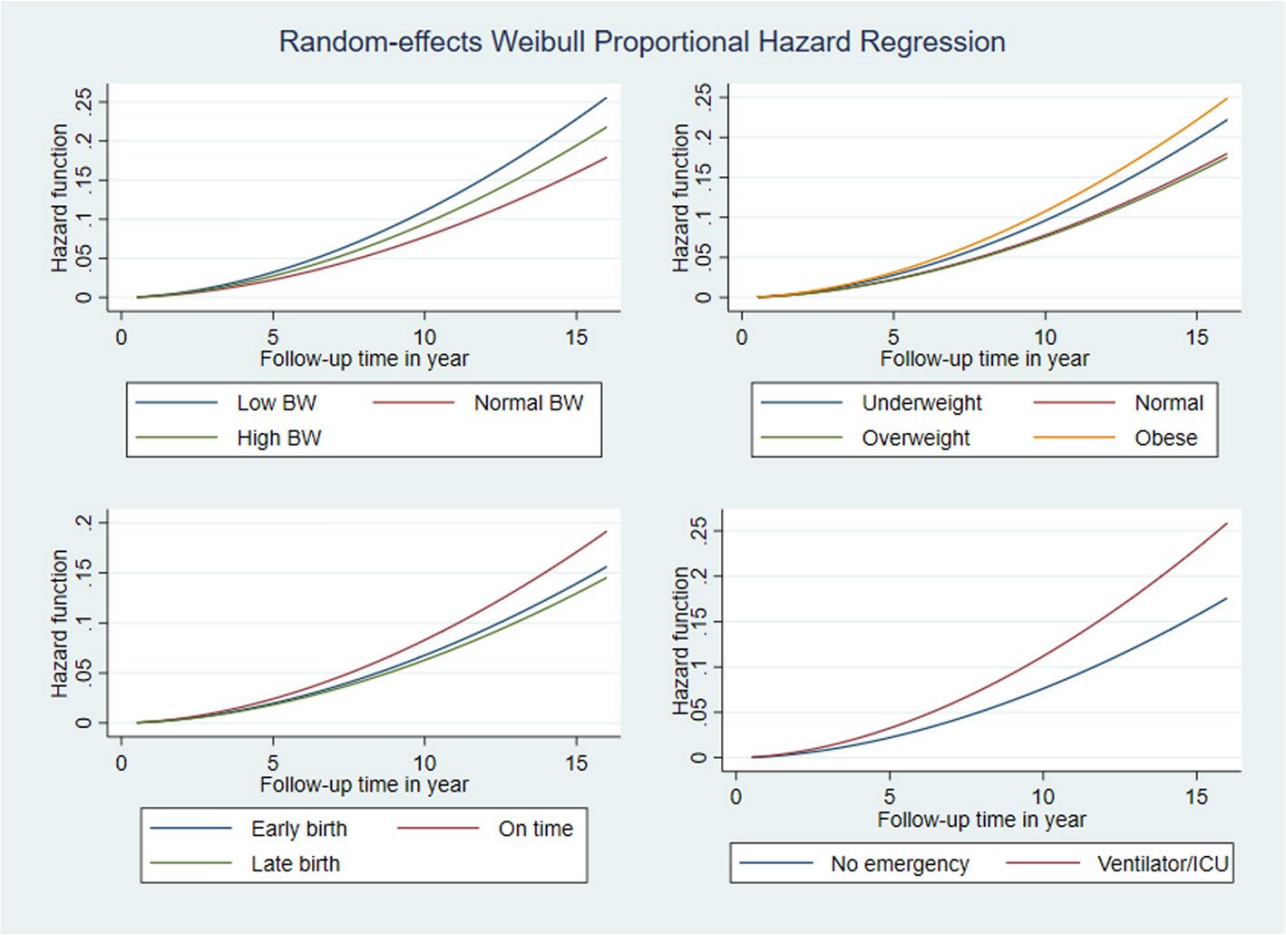
Smooth curves of proportional hazard regression on hazard functions of children across the years by birthweight, obesity status, gestational age and after birth emergency of the children

**Fig. 3:**
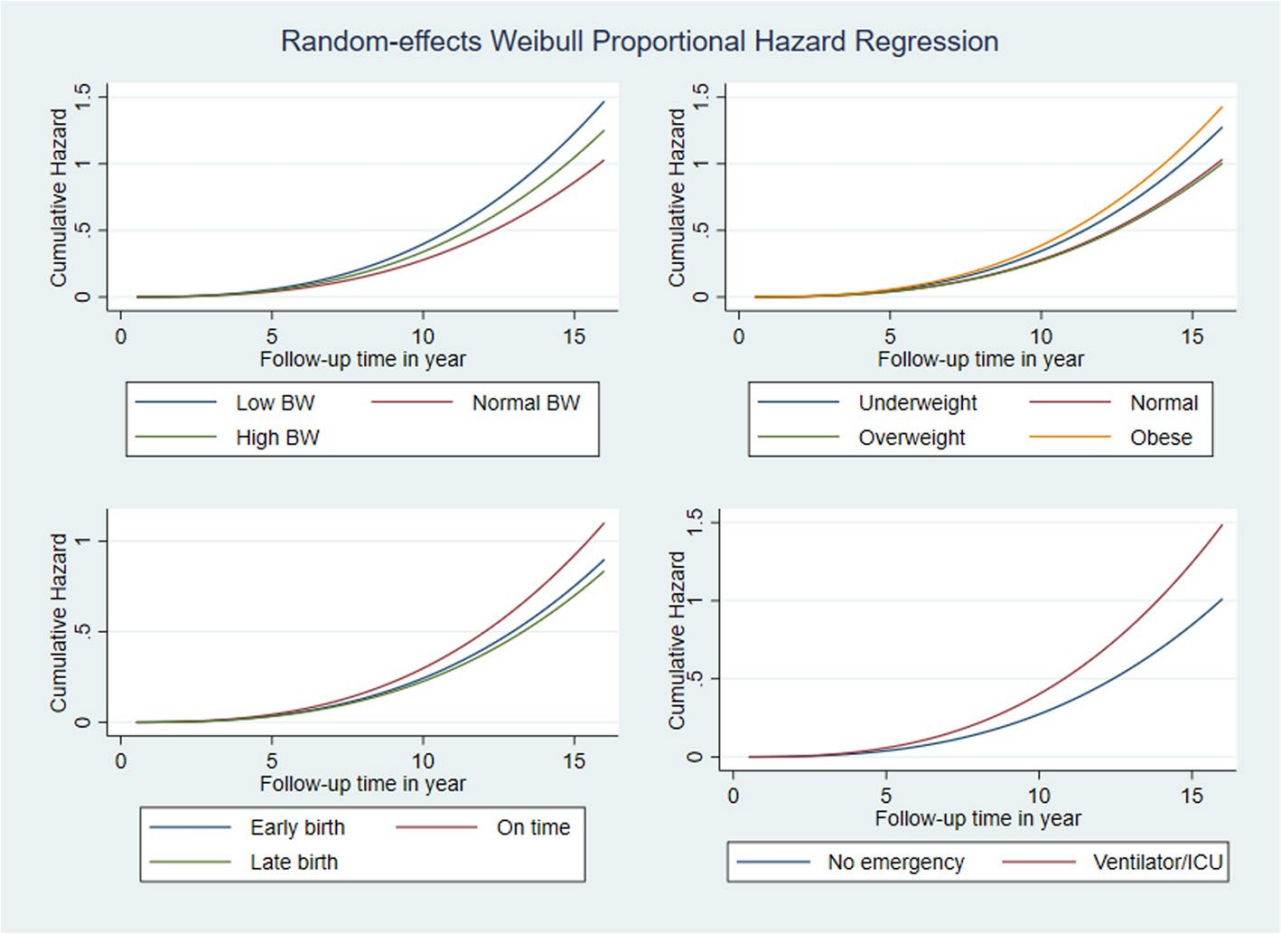
Smooth curves of proportional hazard regression on cumulative hazard functions of children across the years by birthweight, obesity status, gestational age and after birth emergency of the children

**Fig. 4:**
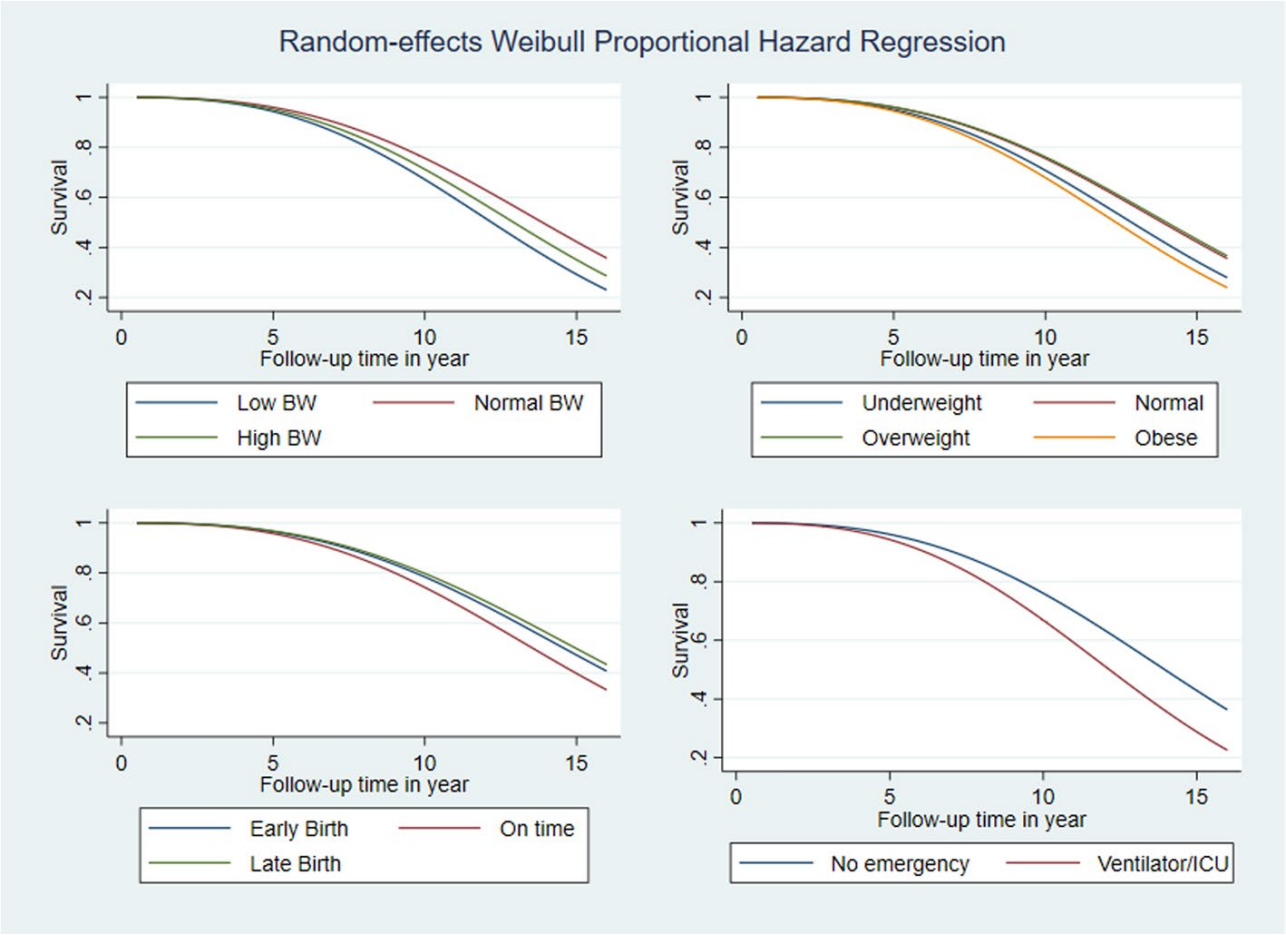
Smooth curves of proportional hazard regression on survival functions of children across the years by birthweight, obesity status, gestational age and after birth emergency of the children

Figure 2 displays random effects Weibull proportional hazard regression curve on hazard ratio over time by birthweight, obesity status, gestational age and after birth emergency of the children. It is evident from the graph that hazard ratio for all risk factors increases over time. The increasing trend was higher for children with low birthweight, obese children and those who received ventilation or ICU services compared to other reference categories shown in Fig. 2. Figure 3 shows the cumulative hazard ratio and Fig. 4 shows the survival rate over time for the same risk factors shown in Fig. 2. Cumulative hazard ratio curves of Fig. 3 have the similar trends of hazard ratio curves shown in Fig. 2, revealing with a steeper increase while follow-up time advances. In Fig. 4, diminishing trends of survival are evident with lower survival rates for low birthweight, obese and emergency service recipient children compared to the children of reference categories.

### Regression model results

Table 3 presents the results of the panel regression model for the hazards of any medical condition or disability among the children based on survival analysis. In the adjusted regression model, low birthweight and obesity were predictive of medical condition or disability, presenting a risk of 1.43 times (Hazard Ratio: 1.43, 95% Confidence Interval: 1.05–1.94) than those with normal birthweight. The time to event analysis also found that obese children were and 1.38 times (HR: 1.38, 95% CI: 1.07–1.79) more likely to have the hazard of a medical condition or disability compared to the normal weight children. However overweight was not significant to predict the medical condition or disability of the children. Among the other independent factors, male children and children who received after-birth emergency services were strongly associated with higher risk of any medical condition or disability with hazard ratio of 1.47 (95% CI: 1.23–1.75) and 1.30 (95% CI:1.14–1.48) respectively (*p*-value < 0.001).

**Table 3:** Panel data parametric survival model for any medical condition/disability health hazard among Australian children, 2004–2018

	Regression Model		
	Hazard ratio	*p-value*	95% CI
**Birthweight**			
2500—4000 grams (ref.)			
< 2500 grams	**1.43**	**0.023**	**1.05–1.94**
4000 + grams	1.22	0.036	1.01–1.46
**Obesity of children**			
Normal weight (ref.)			
Underweight	1.23	0.117	0.95–1.61
Overweight	0.97	0.766	0.81–1.17
Obese	**1.38**	**0.013**	**1.07–1.79**
**Gestational Age**			
Matured birth, 37–42 weeks (ref.)			
Early birth, < 37 weeks	0.83	0. 190	0.60–1.11
Late birth, 42 + weeks	0.76	0.132	0.53–1.09
**After birth emergency**			
No emergency (ref.)			
Ventilation/Intensive Care Unit required	**1.47**	**0.000**	**1.23–1.75**
**Gender of the child**			
Female (ref.)			
Male	**1.30**	**0.000**	**1.14–1.48**

The regression model was adjusted for age of the children at each wave, sex of the children, language spoken at home, indigenous status, whether the study child has both parents, mothers age at birth, education of mothers, and region of residence

## Discussion

The present study focused on Australia and determined the incidence of any medical condition or disability among children based on time to event analysis and investigated its association with child health-related characteristics. The estimated incidence of any medical condition or disability was 26.13 per 1000 person-year among Australian children. The study also found that the hazard rate was higher for children born with low weight or receiving after-birth emergency services.

The main point revealed from the study is that children with low birthweight have higher hazards of developing any medical condition or disability compared to the children who have healthy birthweight. A follow-up study conducted in the USA aligns with our study findings that low birthweight increases children’s risk of any medical condition [19]. In addition, similar results have been observed in some previous studies [7, 8, 10]. However, the results contradict an earlier study that revealed no significant association between low birthweight and subsequent disorder in children [20]. One possible reason could be that low birthweight generates complications for children’s health because it is hard to gain weight and fight infectious diseases [21]. Immaturities in different organs, lower immunity, weakness, and low body fat are other plausible reasons for disease burden due to low birthweight [21].

The hazard of experiencing any medical condition or disability is higher for obese children compared to children who have normal weight. Our findings have added further insight into the existing literature which is consistent with this result [6, 22–24]. A previous Australian study also identified obesity as a risk factor of acquiring a disability [25]. The possible reason could be defined by low physical activity and metabolism deformity among the children who face health problems due to obesity [24, 26].

The children who use emergency services after birth, such as the services of ventilation in the intensive care unit, have a greater risk of living with disabilities in childhood. This agreement equates to an extant study that reported a significant association between ventilation utilisation and health complications [27]. The probable causes might be for difficulties and inconsistencies in perinatal events as this situation increases various health hazards including respiratory and brain injury related health issues [28]. Further, existing research shows that majority of the children in ICU are premature and low birthweight [29–31]. Emergency care provides instant remedies; however, listed reasons create further medical issues among children, which are hard to deal with and have long term consequences [32, 33].

Our study did not find evidence that gestational age has a statistically significant influence on children’s medical condition. However, some retrospective cohort studies have identified it as a contributing factor of disability [7, 34]. Therefore, a further large-scale investigation is required to examine the disparities across the studies through a systematic review.

Our study reveals risk factors of children’s medical condition or disability that might contribute to inventing conservative approaches for public and private organisations to control the children’s medical conditions or disabilities in Australia. First, screening programs based on nutritional and physical activity measurement for children could be planned to tackle the obesity epidemic. For example, each child could receive a diet chart and physical activity schedule considering their body mass index (BMI) through science-based intervention. According to the Australian Health and Wellbeing Strategic Framework 2017 to 2026, these proposed strategies create awareness about disease burden and provide knowledge about the necessity of maintaining a healthy lifestyle [35]. Second, accessible medical interventions are needed for pregnant women that promote understanding of pregnancy-related hazards and health checkup advantages. It might help women to have increased awareness of their health status during the gestation period, increasing the possibility of delivering healthy infants. Third, different health strategies for male children might be designed to provide special attention since birth to lessen disease severity.

This study adds further insights in the current literature. This is the first study identifying the association between infant and child health characteristics with any medical condition or disability among Australian children. Additionally, the study considers 15 years of follow-up data from a large, nationally representative children’s birth cohort in Australia. To escape bias, ‘any medical condition or disability’ is constructed to mean all possible diseases that occurred in childhood because the variability nature of measurement is observed across the studies [36]. Further, a wide range of infant and child health characteristics is included in this cohort study after considering the effect of child health related problems.

This study also has limitations that should be acknowledged. First, examining the causal relationship is not possible due to the unbalanced longitudinal data. Therefore, future study is needed to identify the underlying association by considering the cause and generalizing the association for external settings in Australia. Second, the responses on long term medical conditions or disabilities, the outcome variable of this study, were provided by the respondents, who were either caregivers of the study children or the children themselves, if their age were 14 years or over. The study assumes that the medical conditions or disabilities reported by the respondents were identified or treated by a General Practitioner or specialist earlier, in the context of Australian health-care system. However, the self-reported nature of the outcome variable might lead to reporting bias in the study. Third, disparities among the statistics might have been found as the non-participants made the sample sizes unequal in the waves.

## Conclusion

The current study assessed from a contemporary birth cohort of Australian children that low birthweight, receiving emergency hospital services just after birth, being male and being obese during childhood up to the age of 15 years are associated with increased hazard of having any medical condition or disability. These findings suggest that infants with low birth weight, children who have accessed hospital emergency services and children with obesity need further healthcare monitoring support from both private and public providers to improve the health and wellbeing of Australian children.

## Data Availability

The data used for the study were collected from the Longitudinal Study of Australian Children Dataverse of National Centre for Longitudinal Data. Those interested in accessing this data should contact the Longitudinal Study of Australian Children Dataverse of National Centre for Longitudinal Data, Australia. There are some restrictions on the use of this data and the data application’s approval is subject to a signed confidentiality deed.

## Abbreviations

HR: Hazard Ratio
LSAC: Longitudinal Study of Australian Children
ICU: Intensive Care Unit
BMI: Body Mass Index
